# Cell-penetrating TLR inhibitor peptide alleviates ulcerative colitis by the functional modulation of macrophages

**DOI:** 10.3389/fimmu.2023.1165667

**Published:** 2023-05-05

**Authors:** Bikash Thapa, Seongwon Pak, Dohyeon Chung, Hye Kyoung Shin, Seong Ho Lee, Keunwook Lee

**Affiliations:** ^1^ Institute of Bioscience & Biotechnology, Hallym University, Chuncheon, Republic of Korea; ^2^ Department of Biomedical Science, Hallym University, Chuncheon, Republic of Korea; ^3^ R&D Center, Genesen Co., Ltd, Seoul, Republic of Korea

**Keywords:** toll-like receptors, cell-penetrating TLR inhibitor peptide, ulcerative colitis, macrophage, type 17 helper T cells

## Abstract

Toll-like receptors (TLRs) have a crucial role not only in triggering innate responses against microbes but in orchestrating an appropriate adaptive immunity. However, deregulated activation of TLR signaling leads to chronic inflammatory conditions such as inflammatory bowel disease (IBD). In this study, we evaluated the immunomodulatory potential of a TLR inhibitor in the form of a cell-penetrating peptide using an ulcerative colitis animal model. A peptide derived from the TIR domain of the TLR adaptor molecule TIRAP that was conjugated with a cell-penetrating sequence (cpTLR-i) suppressed the induction of pro-inflammatory cytokines such as TNF-α and IL-1β in macrophages. In DSS-induced colitis mice, cpTLR-i treatment ameliorated colitis symptoms, colonic tissue damage, and mucosal inflammation. Intriguingly, cpTLR-i attenuated the induction of TNF-α-expressing proinflammatory macrophages while promoting that of regulatory macrophages expressing arginase-1 and reduced type 17 helper T cell (Th17) responses in the inflamed colonic lamina propria. An *in vitro* study validated that cpTLR-i enhanced the differentiation of monocyte-driven macrophages into mature macrophages with a regulatory phenotype in a microbial TLR ligand-independent manner. Furthermore, the cocultivation of CD4 T cells with macrophages revealed that cpTLR-i suppressed the activation of Th17 cells through the functional modulation of macrophages. Taken together, our data show the immunomodulatory potential of the TLR inhibitor peptide and suggest cpTLR-i as a novel therapeutic candidate for the treatment of IBD.

## Introduction

The gastrointestinal barrier faces the challenge of co-existing with the microbial community. An agitated homoeostasis of the mucosal immunity with commensal microbes or by infection with pathogens is implicated in mucosal inflammation, leading to chronic conditions such as inflammatory bowel diseases (IBD) (1). Toll-like receptors (TLRs) as sensors of a microbe-associated molecular pattern are involved in the pathogenesis of IBD not only by maintaining the mucosal homeostasis but protecting against pathogen infection (2). Indeed, *Tlr4* ^-/-^ and *Myd88* ^-/-^ mice are less sensitive to DSS-induced colonic inflammation, which could be due to the diminished immune responses to microbiota (3). Similarly, depletion of MyD88 prevents spontaneous colitis caused by *Il10* deficiency and chronic inflammation in response to commensal microbes (4). In contrast, TLR9 deficient mice are more susceptible to DSS-induced colitis and administration of TLR9 agonist alleviates colitis, suggesting a protective role against intestinal barrier damage (5, 6). Consistently with studies obtained from experimental IBD models, aberrant expression and genetic variations in TLR genes are reported to be highly associated with the risk and pathogenesis of IBD such as Crohn’s disease and ulcerative colitis (7). In this context, targeting TLR signaling would be a potential strategy for the treatment of IBD.

TLRs share common adaptor molecules, enabling the intracellular Toll/IL-1 receptor (TIR) domain of the receptors to induce the activation and nuclear translocation of transcription factors such as NF-κB and interferon regulatory factors (8, 9). The dimerization of TLRs recruits cytosolic TIR domain-containing adaptor protein (TIRAP) or TIR-containing adaptor-inducing interferon β (TRIF)-related adaptor molecules (TRAM), leading to a subsequent interaction with MyD88 and TRIF, respectively (8). Thus, this complex formation with the intracellular domain of TLRs in a homologous manner with different TIR domain-containing adaptor molecules is critical for linking the TLRs and the outcome of the immune responses. In addition to blocking the binding of TLR ligands to the receptor, interfering with the interaction between TLRs and adaptor molecules has been shown to be beneficial to inflammatory conditions and autoimmune diseases (10). TAK-242 is a small molecule inhibitor of TLR4 that disrupts the interaction of the TIR domain with TIRAP and TRAM (11), and mitigates sepsis induced by endotoxins and polymicrobial peritonitis (12, 13). Moreover, TAK-242 treatment was shown to alleviate experimental autoimmune myositis by suppressing the induction of IFN-γ and IL-17A (14). Studies reported that peptides as a competitive inhibitor for the TLR4 adaptors ameliorated LPS-induced sepsis, collagen-induced arthritis and systemic lupus erythematosus in mouse models (15, 16). Of note, Jung et al. reported that specifically targeting TLR2 by a peptide derived from the trans-membrane domain was sufficient to reduce the severity of chemically induced colitis by the suppressing the proinflammatory activation of macrophages (17). These studies highlight the therapeutic potential of peptide inhibitors of TLR signaling in IBD.

Macrophages in the intestinal mucosa are essential for homeostatic functions such as clearance of cell debris and immune surveillance. Upon mucosal tissue damage or by pathogen infection, macrophages become activated into a proinflammatory phenotype, leading to inflammation and tissue damage (18). In addition to the conventional macrophage polarization paradigm, recent studies revealed the “monocyte-macrophage waterfall” in the gut lamina propria where there is a gradual differentiation process of macrophages (19). Circulating monocytes infiltrate into the intestinal mucosa and give rise to macrophages with a tolerogenic resident phenotype via macrophages with a high proinflammatory potential, which might be arrested during gut inflammation (19, 20). This model supports the idea of functional plasticity between classically activated macrophages (M1) and alternatively activated macrophages (M2), and the trans-differentiation of macrophages with the M1 phenotype to M2 cells might be critical for resolving intestinal inflammation. Furthermore, macrophages have a role in the regulation of T cells by presenting costimulatory ligands (*e.g.*, CD80/86 and PD-L1/2) and secreting immune modulators (*e.g.*, IL-1β, IL-12, IFN-β, and arginase) (21–24). Intestinal monocytes/macrophages derived from patients with IBD promote polarization of type 1 and type 17 helper T cells rather than inhibiting CD4 T cell proliferation as resident tolerogenic macrophages do (25, 26). Thus, targeting the functional activation and differentiation of macrophages offers an effective therapeutic intervention in mucosal inflammation.

In this study, we designed a synthetic peptide derived from the TIR domain of the TLR adaptor TIRAP conjugated with a cell-penetrating peptide that permits the intracellular delivery of hydrophilic cargo peptides (27, 28). The inhibition of TLR signaling by the cell-penetrating TLR antagonist peptide suppressed the proinflammatory activation of macrophages and ameliorated colitis in an ulcerative colitis model. Moreover, we provided mechanistic insight showing that the TLR inhibitor peptide impedes colonic inflammation by modulating the functional responses of macrophages and the induction of type 17 helper T cells.

## Results

### Cell-penetrating TLR inhibitor peptide suppresses the proinflammatory activities of macrophages

**Figure 1:**
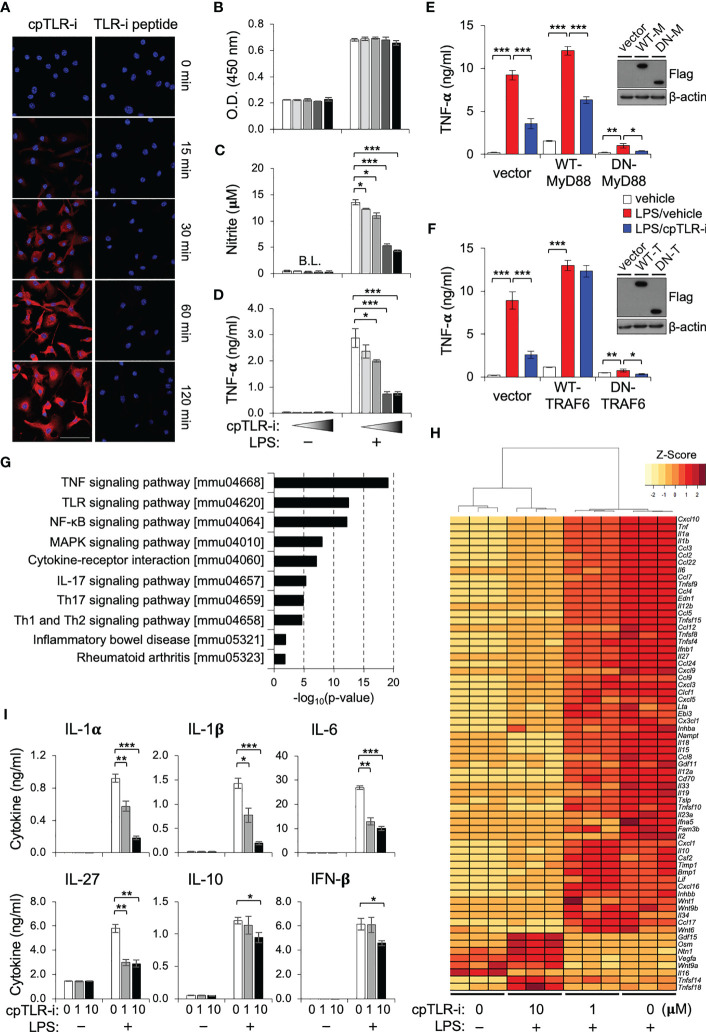
cpTLR-i suppresses the proinflammatory responses of macrophages. **(A)** BMDMs were incubated with 2 μM cpTLR-i or a control TLR-i peptide lacking the cell-penetrating sequence (TLR-i) for the indicated times. Shown are the representative confocal images stained with antibody against the TLR inhibitor peptide (red) and DAPI (blue). BMDMs pretreated with cpTLR-i (0, 0.08, 0.4, 2 and 10 μM) were cultured in the presence or absence of LPS. **(B, C)** Cell viability and NO production were determined 24 h after LPS activation. **(D)** Amounts of TNF-α in the culture supernatant were measured by cytokine beads array (CBA): *, p < 0.05; ** p < 0.01, *** p < 0.001. **(E, F)** RAW 264.7 cells were transfected with plasmids expressing wild-type (WT) or dominant negative (DN) forms of MyD88 and TRAF6, respectively. Transfected cells pretreated with 2 μM cpTLR-i were activated with LPS for 8 h and secretion of TNF-α was determined by CBA. Ectopic expression of the indicated proteins was confirmed by Western blotting. **(G, H)** BMDMs pretreated with cpTLR-i (1 and 10 μM) were activated with LPS for 2 h and the differentially expressed genes were analyzed by RNA sequencing. **(G)** Significantly enriched signaling pathways in cpTLR-i-treated samples in comparison to those activated with LPS alone were determined by the KEEG Pathway Analysis. **(H)** The heatmap for genes encoding cytokines, chemokines and immune modulators is shown with a hierarchical cluster analysis. **(I)** BMDMs pretreated with cpTLR-i (0, 1, 10 μM) were cultured in the presence or absence of LPS for 8 h and cytokine secretion was quantitated by CBA: n = 6.

Upon infection or tissue damage, macrophages experience a wide range of extracellular stimuli that include TLR ligands and trigger inflammatory responses. For modulation of intracellular signaling downstream from TLRs, we designed a cell-penetrating peptide for which a conserved βC motif peptide of the TIR domain found in the TLR receptor adaptor TIRAP was conjugated with an 18-mer amphipathic model peptide, one of the cell-penetrating sequences as an efficient transport for different peptide cargoes (27, 28). After treating bone marrow-derived macrophages (BMDMs) with the cell-penetrating TLR inhibitor peptide (cpTLR-i), we observed intracellular translocation of the peptide in the presence of the cell-penetrating sequence (**Figure 1A**). Treatment with brefeldin A or rotenone did not impede the translocation of cpTLR-i into BMDMs, suggesting an endocytosis- and ATP-independent transduction of the peptide (**Supplementary Figure S1A**). The cell penetrating abilities of cpTLR-i into macrophages, B cells, T cells and colorectal cancer cell SW480 were comparable (**Supplementary Figure S1B**). cpTLR-i inhibited the induction of nitric oxide (NO) and the secretion of proinflammatory cytokines such as TNF-α from the BMDMs activated with LPS in a dose-dependent manner without affecting the cell viability (**Figures 1B–D**). However, control βC motif peptide lacking the cell-penetrating conjugate (TLR-i peptide) and the cell-penetrating peptide (CP peptide) alone did not inhibit LPS-induced production of NO and TNF-α (**Supplementary Figures S1C–E**). These results indicate that the anti-inflammatory potential of the TLR inhibitor depends on the cell-penetrating ability of the peptide.

To elucidate the functional impact of cpTLR-i on macrophages, we performed an RNA-seq analysis using the BMDMs. Pathway analysis revealed that most genes affected by cpTLR-i were related to inflammatory signaling downstream from TLRs including the NF-κB and MAPK pathways, which implies a competition of cpTLR-i with proximal adaptor molecules (*e.g.*, TIRAP and MyD88) to TLR receptors (**Figure 1G**). As expected (29, 30), ectopic expression of TRAF6, a signaling adaptor downstream from MyD88 restored LPS-induced TNF-α production in the presence of cpTLR-i, whereas that of the proximal adaptor MyD88 did not (**Figures 1E, F**). Of note, the effect of cpTLR-i was significantly related to type 17 immune responses and implicated in inflammatory bowel disease (IBD). The transcriptome of BMDMs displayed marked changes in genes encoding proinflammatory cytokines and chemokines by the cpTLR-i treatment as shown by the heat map in **Figure 1H**. Specifically, LPS induction of genes involved in IL-17 signaling (*Ccl2, Cxcl10, ll1b, IL6* and *Csf2*), Th17 cell differentiation (*Il1b, Il27, Il6* and *Il23a*), and IBD (*IL1a, Il1b, Il12b, IL6* and *Il18*) was restrained in a dose-dependent manner (**Figure 1H**, **Supplementary Figure S1F**). On the other hand, treatment with a higher concentration of cpTLR-i (10 μM) upregulated the expression of growth factors (*Osm*, *Gdf15* and *Vegfa*) that are associated with the functional responses of the alternatively activated macrophages (31–33). We also validated that cpTLR-i suppressed the LPS-induced secretion of Th17-promoting cytokines including IL-1α, IL-1β, and IL-6 at the protein level (**Figure 1I**). LPS induction of IL-10 and IFN-β was not affected by 1 μM cpTLR-i but was attenuated with a higher concentration (10 μM) (**Figure 1I**). Based on these transcriptomic analyses, we next investigated the therapeutic potential of cpTLR-i using an animal model associated with Th17-mediated gut inflammation (34).

### cpTLR-i ameliorates colitis and the colonic mucosal inflammation induced by DSS

**Figure 2:**
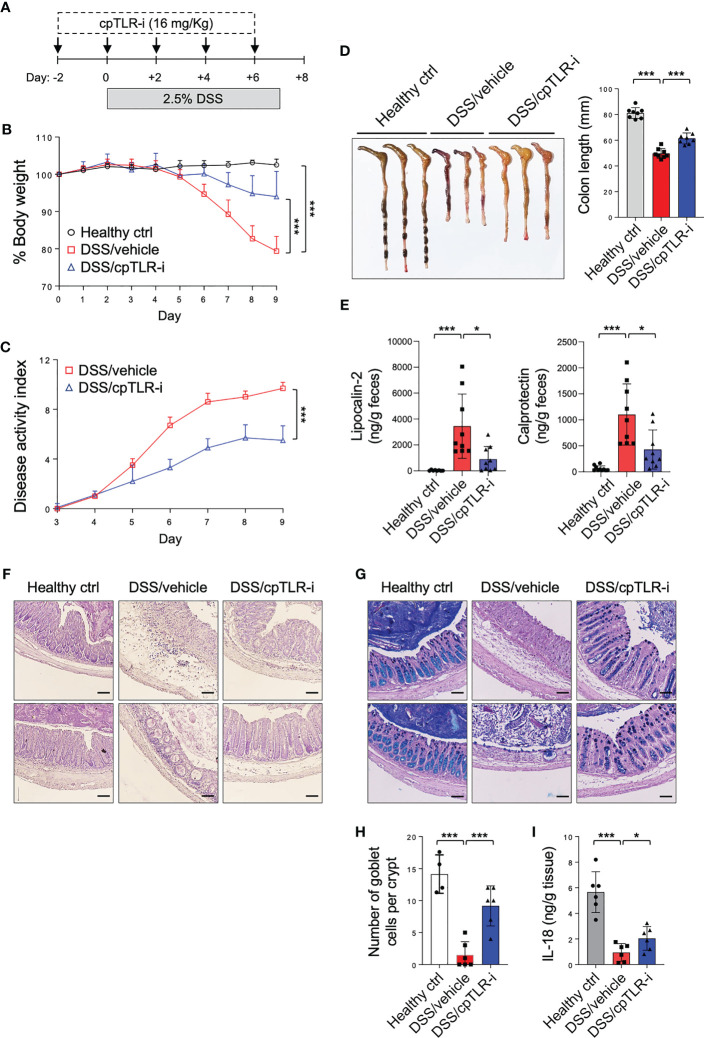
cpTLR-i alleviates DSS-induced colitis. **(A)** Experimental scheme of DSS administration with cpTLR-i treatment is shown. **(B)** Body weight was measured daily and represented as a mean percentage of body weight. **(C)** Disease activity index was scored as describe in “Material’s and Methods”. Results are the representative of three independent experiments obtained from 10 mice per group. P values among different groups were analyzed by 2-way ANOVA with Bonferroni correction: ***, p < 0.001. **(D)** The colons were isolated from the mice at day 9 and their lengths were measured. **(E)** Levels of lipocalin-2 and calprotectin in the feces at day 5 were quantitated by ELISA. **(F, G)** Shown are the representative histology images of the distal colons tissues stained with H&E **(F)** and PAS-Alcian Blue **(G)**. **(H)** Number of goblet cells per crypt in the distal colons. **(I)** The distal colon explants were cultured and ELISA was performed with the culture supernatants. Amount of IL-18 was normalized to the weight of each colon piece. *p < 0.05.

The potential of cpTLR-i to inhibit intracellular TLR signaling in gut inflammation was determined in mice given DSS in drinking water. Mice were injected with cpTLR-i or control peptides along with DSS administration shown in **Figure 2A**. Treatment with the control TLR-i peptide lacking the cell-penetrating conjugate or the CP peptide alone did not affect the colitis symptoms, which include body weight loss, diarrhea, rectal bleeding, and reduced colon length (**Supplementary Figure S2**). In contrast, cpTLR-i treatment ameliorated colitis shown by significant decreases in the body weight loss, disease activities and colon length shortening caused by the DSS administration (**Figures 2B–D**). We also observed reduced amounts of fecal inflammatory factors such as lipocalin-2 and calprotectin in the feces of mice treated with cpTLR-i (**Figure 2E**). Histological analysis of the colonic tissues revealed that cpTLR-i mitigated not only epithelial architectural damages but also the infiltration of inflammatory cells within the colonic mucosa (**Figure 2F**). Loss of goblet cells, specialized epithelial cells that secret mucins, is a hallmark of ulcerative colitis, and IL-18 is important for the breakdown of the mucosal barrier integrity (35). Consistently with the reduced expression of *Il18* observed in the transcriptome analysis (**Figure 1H**), we found a profound diminution in the loss of goblet cells in the colon of mice treated with cpTLR-i in relation to decreased secretion of IL-18 from the colonic tissue explants (**Figures 2G–I**). These data suggest a therapeutic potential of cpTLR-i in the management of colitis.

**Figure 3:**
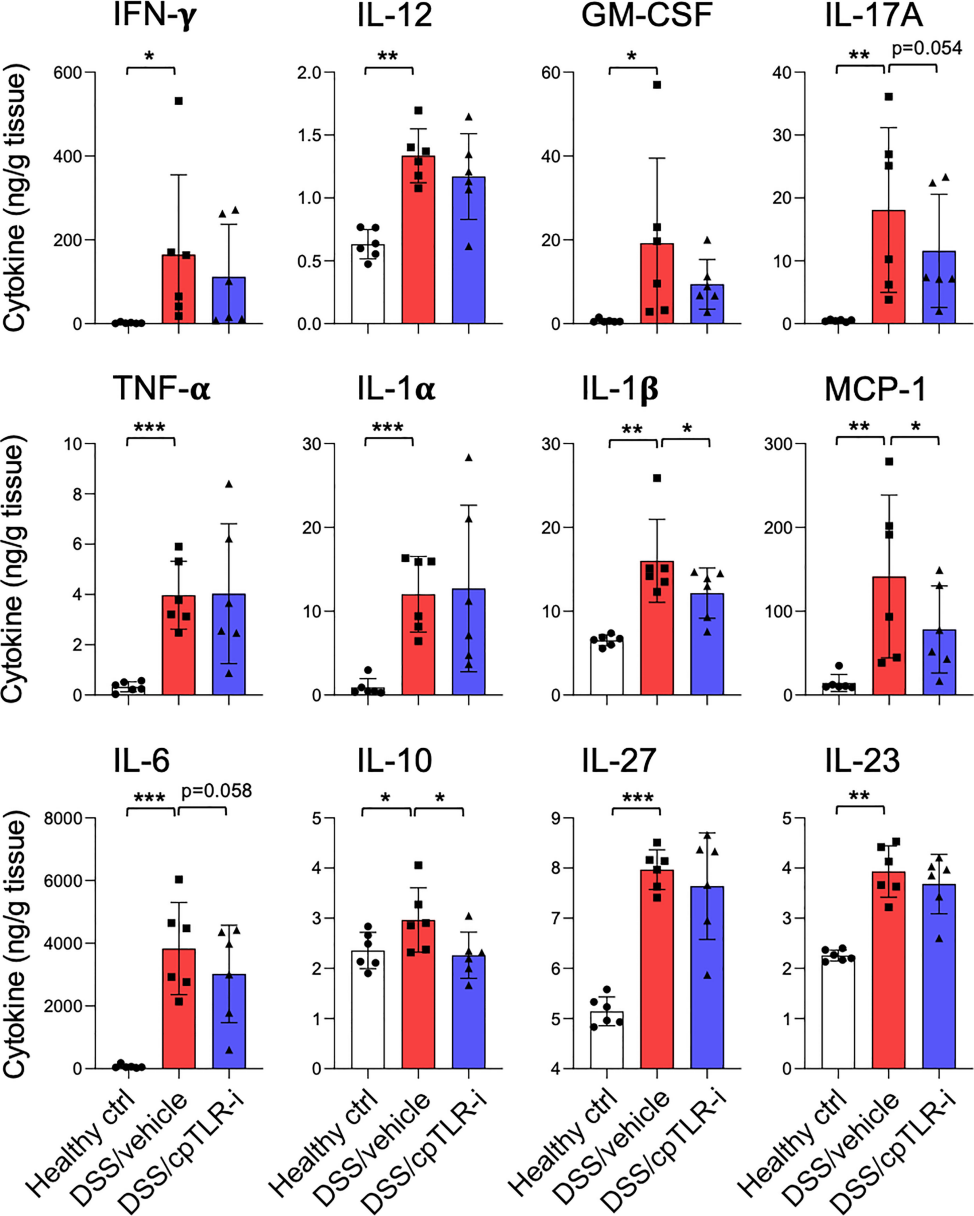
Effect of cpTLR-i on the secretion of inflammatory cytokines from the *ex vivo* colon explants. The colons were isolated from the colitogenic mice as in **Figure 2** and pieces of the distal colons were plated in a 24-well plate. 24 h after the *ex vivo* colon explants culture, CBA were performed with the culture supernatants. Amount of each cytokine was normalized to the weight of each colon piece: n = 6. *p < 0.05; **p < 0.01; ***p < 0.001.

The anti-inflammatory effect of cpTLR-i was further validated by examining the cytokine profiles secreted from the distal colon tissue explants (**Figure 3**). Proinflammatory cytokines associated with Th17 responses (IL-1β, IL-6, IL-17, IL-23, and GM-CSF) and those of Th1 responses (IL-12, IFN-γ and TNF-α) were highly upregulated in mice given DSS. Importantly, cpTLR-i treatment resulted in a significant decrease in the induction of IL-1β in the inflamed colonic tissues, while only affecting marginally other Th17-related factors that include IL-6 and IL-17A. Secretion of Th1 cytokines such as IFN-γ and IL-12 from the colon explants was not decreased by the cpTLR-i treatment (**Figure 3**). In parallel to the attenuated inflammation observed in the histological analysis, the anti-inflammatory cytokine IL-10 was produced less in the colon explants of mice treated with cpTLR-i. Collectively, these results underline the potential of cpTLR-i to abrogate the intracellular TLR signaling pathways in protection against colonic inflammation and mucosal tissue damage in colitis.

### cpTLR-i attenuates activation of type 17 helper T cells and modulates inflammatory responses of macrophages in the inflamed colon

**Figure 4:**
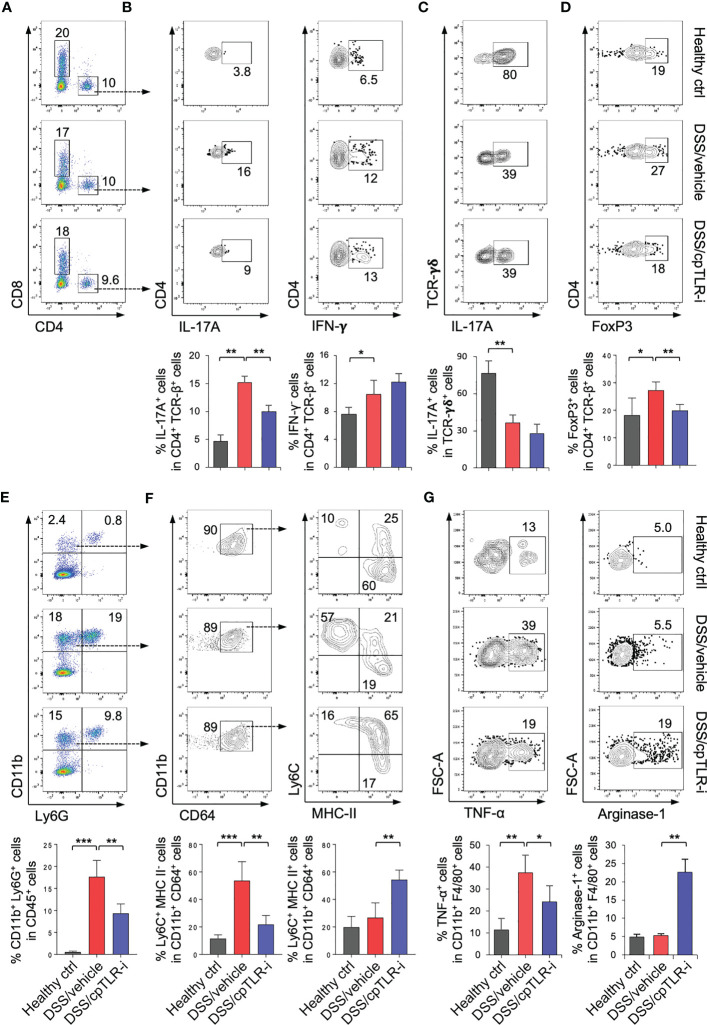
cpTLR-i attenuates Th17 cell responses and modulates macrophages in the inflamed colon. Lamina propria cells in the colonic tissues were isolated from the colitogenic mice and analyzed by flow cytometry. **(A)** Representative FACS profiles with prevalence of CD4^+^ and CD8^+^ T cells are shown in the viable CD45^+^ cell gate. **(B)** Expression of IL-17A and IFN-γ in CD4 T cells were measured by intracellular cytokine staining. Percentages of CD4 T cells expressing IL-17A and IFN-γ are illustrated as bar graphs below the FACS plots: n = 5. **(C)** IL-17A^+^ γδ T cells was analyzed in the CD45^+^ CD3ϵ ^+^ TCRγδ^+^ cell gate. **(D)** Expression of FoxP3 in the viable CD4 T cell gates and prevalence of FoxP3^+^ regulatory T cells were determined by FACS. **(E)** Representative FACS profiles of CD11b and Ly6G in the viable CD45^+^ cell gate and percentage of CD11b^+^ Ly6G^+^ granulocytes are shown. **(F)** CD11b^+^ Ly6G^-^ CD64^+^ cells were further analyzed into subpopulations based on expression Ly6C and MHC-II, namely Ly6C^+^ MHC-II^-^, Ly6C^+^ MHC-II^+^ and Ly6C^-^ MHC-II^+^ cells. Prevalence of subpopulations of CD11b^+^ Ly6G^-^ CD64^+^ monocytes/macrophages are illustrated as bar graphs. **(G)** Expression of TNF-α and Arginase-1 in the CD11b^+^ Ly6G^-^ CD64^+^ cells were determined by intracellular staining: n = 5. *p < 0.05; **p < 0.01; ***p < 0.001.

To determine the effect of cpTLR-i on the infiltration and functional responses of immune cell populations in the inflamed colonic mucosa, we isolated cells from the colonic lamina propria and analyzed them by flow cytometry. As expected (34), we observed a profound induction of IL-17-producing type 17 T (Th17) cells and IFN-γ-expressing type 1 T (Th1) cells in the inflamed colonic tissue, and increased infiltration of CD4^+^ T cells (**Figures 4A, B**; **Supplementary Figure S3**). cpTLR-i treatment resulted in a significant reduction in IL-17^+^ Th17 cells within the colonic mucosa as shown in **Figure 4B**. On the other hand, IFN-γ^+^ Th1 cells in the inflamed colonic mucosa were not decreased by cpTLR-i. γδ T cells, one of the most abundant immune populations in the mucosal epithelium, are important for maintenance of the intestinal barrier integrity through the production of IL-17 (36, 37). DSS treatment resulted in decreased prevalence of IL-17^+^ γδ T cells in the colonic lamina propria, which was not affected by cpTLR-i (**Figure 4C**). The percentage of FoxP3^+^ regulatory T cells (Treg), a CD4 T cell subset essential for resolving inflammation, was elevated by DSS and decreased by cpTLR-i treatment (**Figure 4D**). These *in vivo* data support the transcriptomic analysis, indicating a suppressive effect of cpTLR-i on Th17-mediated inflammatory responses. Th17 cells and IL-17 mediate inflammatory responses within the inflamed mucosa leading to the recruitment of immune populations, especially neutrophils, and the production of fecal inflammation mediators such as calprotectin and lipocalin-2 (38, 39). Along with the attenuated Th17 cell activation and decreased fecal inflammation shown in **Figure 2E**, cpTLR-i significantly attenuated the infiltration of CD11b^+^ Ly6G^+^ neutrophils in the colonic tissues (**Figure 4E**; **Supplementary Figure S3**).

Macrophages are abundant immune populations in the colonic mucosa, and monocytes become rapidly recruited in the inflamed colon and repopulated into functionally distinct macrophages in response to the environmental milieu (19, 20). While the majority of Ly6G^-^ CD11b^+^ CD64^+^ populations were Ly6C^-^ MHC-II^+^ macrophages at steady-state, Ly6C^+^ MHC-II^-^ monocytes were substantially increased in the colonic lama propria of mice given DSS (**Figures 4E, F**). Intriguingly, cpTLR-i treatment resulted in a significant reduction in the prevalence of Ly6C^+^ MHC-II^-^ monocytes but an increase in that of Ly6C^hi^ MHC-II^hi^ macrophages, although it did not suppress the infiltration of monocytes/macrophages into the colonic mucosa (**Figure 4F**). This result implies that that cpTLR-i likely regulates the repopulation of macrophages derived from circulating monocytes. Moreover, cpTLR-i attenuated the induction of TNF-α^+^ macrophages whereas enhanced that of Arginase-1^+^ cells (**Figure 4G**), revealing an immunomodulatory potential of cpTLR-i in the functional polarization of macrophages. Therefore, differentiation and/or induction of functionally distinct macrophages could be regulated by selective inhibition of TLR signaling, which contributes to alleviating colitis.

**Figure 5:**
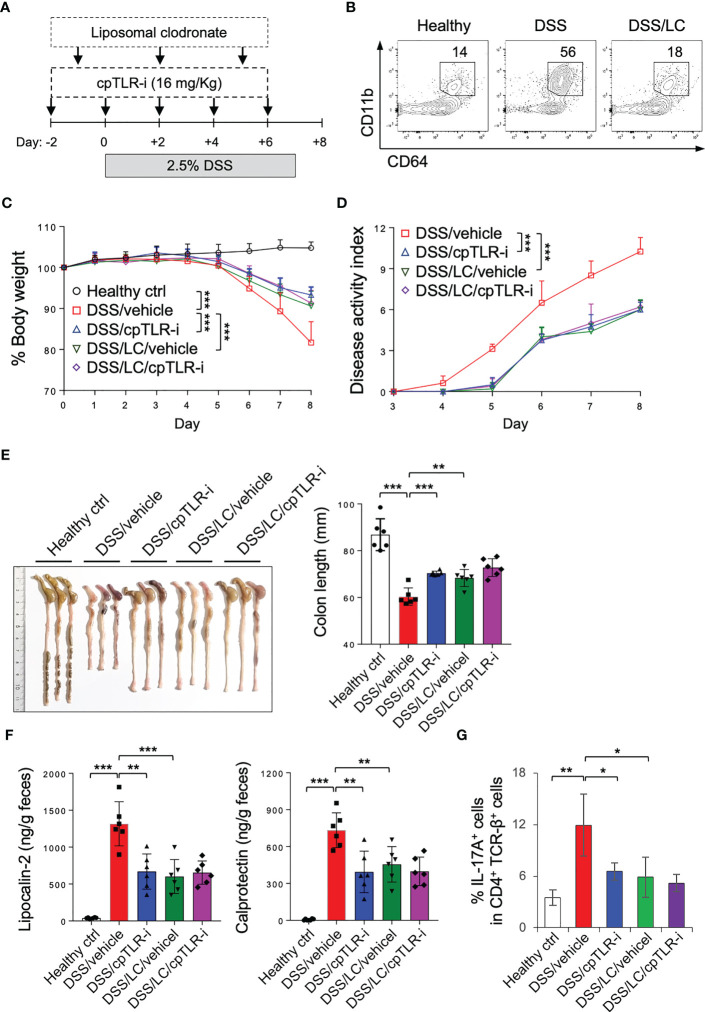
cpTLR-i could not mitigate colitis in mice depleted macrophages. **(A)** Experimental scheme of DSS administration with liposomal chrodronate (LC) treatment is shown. **(B)** Lamina propria cells were isolated from the colon and analyzed by flow cytometry. Representative FACS profiles of CD11b and CD64 in the viable CD45^+^ cell gate are shown. **(C, D)** Body weight and disease activity index were measured as in **Figure 2**: n = 8. P values among different groups were analyzed by 2-way ANOVA with Bonferroni correction. **(E)** The colons were isolated from the mice at day 8 and their lengths were measured. **(F)** Amounts of lipocalin-2 and calprotectin in the feces at day 5 were quantitated by ELISA. **(G)** IL-17A-expressing CD4 T cells in the colonic lamina propria were analyzed by flow cytometry as in **Figure 4B**. *p < 0.05; **p < 0.01; ***p < 0.001.

To further elucidate the impact of cpTLR-i on macrophages during colonic inflammation, we used liposomal clodronate to deplete macrophages (40) (**Figure 5A**). Upon DSS administration, CD11b^+^ CD64^+^ monocyte/macrophage populations were increased in in the colonic lamina propria, which was markedly reduced in mice treated with liposomal clodronate (**Figure 5B**). Consistently with other studies (40–42), macrophage depletion resulted in improved colitis symptoms as shown by decrease in body weight loss, disease activities and colon length shortening (**Figures 5C–E**). Macrophage depletion also led to reduced production of lipocalin-2 and calprotectin in the feces and attenuated induction of Th17 cells in the colonic lamina propria (**Figures 5F, G**). Intriguingly, cpTLR-i did not have a beneficial effect on colitis in mice treated with liposomal clodronate (**Figures 5C–G**). These results indicate an important role of macrophages in triggering colonic inflammation and imply that the therapeutic potential of cpTLR-i depends largely on proinflammatory activities of macrophages.

### cpTLR-i promotes functional maturation of macrophages with a regulatory phenotype

**Figure 6:**
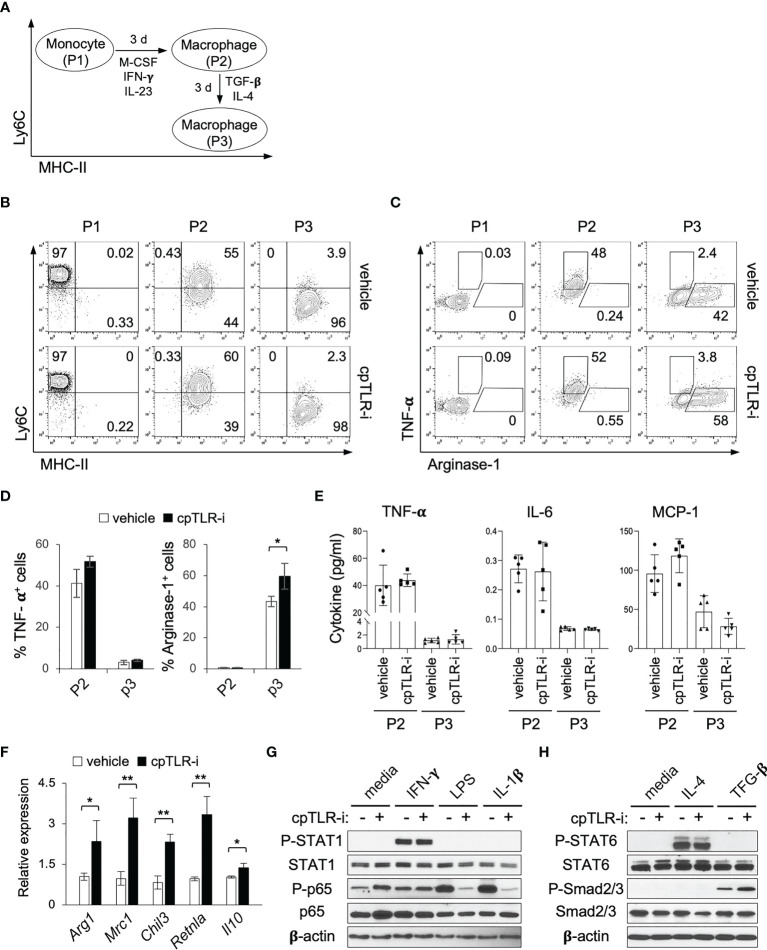
cpTLR-i enhances the induction of macrophages with a regulatory phenotype. **(A)** Scheme of the *in vitro* culture mimicking the monocyte-macrophages waterfall. **(B, C)** P1 monocytes, P2 and P3 macrophages were analyzed by flow cytometry. Shown are the representative FACS profiles in the viable CD11b^+^ CD64^+^ gate from the three independent experiments. **(D)** Percentage of TNF-α^+^ and Arginase-1^+^ cells in the *in vitro* generated P2 and P3 macrophages are illustrated as bar graphs. **(E)** Secretion of TNF-α, IL-6 and MCP-1 from the P2 and P3 macrophages were determined by CBA. **(F)** Expression of the indicated genes in P3 macrophages were analyzed by real-time RT-PCR: n = 4. **(G)** Purified monocytes were activated for 30 min with IFN-γ, LPS or IL-1β in the presence or absence of 5 μM cpTLR-i and analyzed by Western blotting. Immunoblots of β-actin and unphosphorylated proteins were used as a loading control. **(H)** *In vitro* generated P2 macrophages were stimulated with IL-4 or TGF-β in the presence of cpTLR-i and analyzed as in **(G)**. *p < 0.05; **p < 0.01.

cpTLR-i treatment resulted in the decreased prevalence of Ly6C^+^ MHC-II^-^ monocytes and increased Arginase-1^+^ macrophages in the inflamed colonic mucosa (**Figures 4F, G**). To gain further insight into how cpTLR-i regulates monocyte differentiation and functional maturation of macrophages, we used a modified *in vitro* culture that recapitulates the monocyte-macrophage waterfall observed in the inflamed colonic mucosa (**Figure 6A**) (43). When purified Ly6C^hi^ MHC-II^-^ monocytes (P1) were grown in the presence of M-CSF, IFN-γ and IL-23, they gave rise to Ly6C^int^ MHC-II^+^ macrophages (P2) that exhibited an immature proinflammatory phenotype, including the production of TNF-α, IL-6 and MCP-1 (**Figures 6B–E**). Then, when *in vitro* generated P2 macrophages were further cultured with TGF-β and IL-4, they differentiated into Ly6C^-^ MHC-II^+^ cells with a mature regulatory phenotype (P3) such as the expression of Arginase-1 along with the attenuated induction of proinflammatory cytokines including TNF-α (**Figures 6B–E**). When we added cpTLR-i to this *in vitro* culture, we did not observe a significant effect on the differentiation of P1 monocyte into P2 macrophages and on the induction of TNF-α and IL-6 (**Figures 6B–E**). On the other hand, cpTLR-i treatment significantly enhanced the induction of Arginase-1^+^ macrophages although it did not affect the prevalence of Ly6C^-^ MHC-II^+^ P3 cells (**Figures 6B–E**). It was also validated that cpTLR-i upregulated the functional maturation of macrophages shown by the increased expression of *Mrc1, Chil3*, *Retnla* and *Il10*, which is associated with the alternatively activated (“M2”) macrophages (**Figure 6F**) (44). Together, these data show that the abrogation of TLR signaling by cpTLR-i could promote the immunomodulatory potential of macrophages, which might be independent of microbial TLR stimulation.

Because we did not add a TLR ligand to the *in vitro* monocyte culture, we needed to define how cpTLR-i enhanced the functional maturation of P3 macrophages with the regulatory phenotype. cpTLR-i treatment did not affect IFN-γ-induced phosphorylation of STAT1, while it apparently suppressed LPS- or IL-1β-induced phosphorylation of NF-κB p65, a canonically downstream from TLR4 and IL-1 receptor, respectively (**Figure 6G**). It also did not modulate the phosphorylation of STAT6 in P2 macrophages activated with IL-4, a classical M2- polarizing stimulus (**Figure 6H**). Importantly, we observed a significant increase in TGF-β-induced phosphorylation of Smad2/3 when the *in vitro* generated P2 macrophages were treated with cpTLR-i (**Figure 6H**). Transcriptome analysis of BMDMs also validated that the expression of TGF-β target genes that contain Smad2/3 binding sites (*e.g.*, *Id1*, *Smad7* and *Dap2ip*) was upregulated by cpTLR-i whereas that was suppressed by LPS (**Supplementary Figure S4**) (45). This result implies that intracellular signaling pathways downstream from TLRs could negatively regulate TGF-β-receptor-mediated Smad2/3 activation even in the absence of a microbial TLR ligand. Thus, this *in vitro* culture data suggests that the inhibition of TLR signaling by cpTLR-i could upregulate TGF-β-induced functional responses of the regulatory macrophages.

### cpTLR-i impedes activation of Th17 cells through the modulation of macrophages

**Figure 7:**
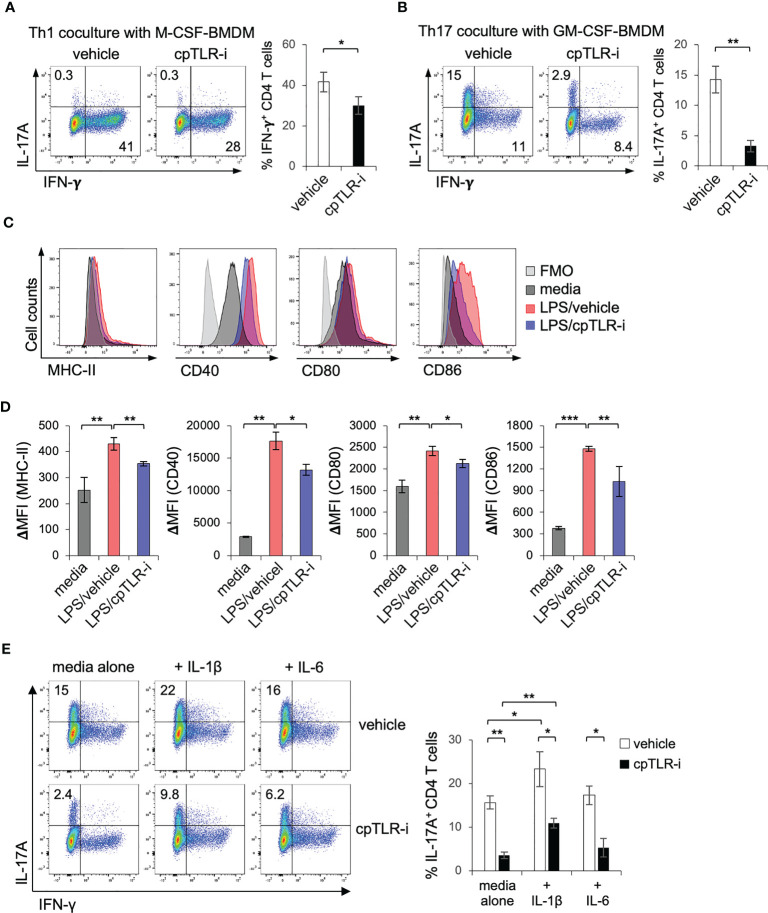
cpTLR-i suppresses induction of Th17 cells through the modulation of macrophages. **(A, B)** BMDMs derived with M-CSF or GM-CSF were activated with LPS in the presence or absence of cpTLR-i for 6 (h) Activated BMDMs were washed and CD4 T cells were cocultured with the BMDMs under Th1 or Th17 polarizing condition. 4 d after the cocultivation, CD4 T cells were stained intracellularly with antibodies against IL-17A and IFN-γ. Shown are the representative FACS profiles in the viable CD4^+^ T cell gate and bar graphs indicating percentages of IL-17A^+^ or IFN-γ^+^ CD4 T cells: n = 3. **(C, D)** BMDMs were pretreated with cpTLR-i and activated with LPS for 24 (h) Cell surface expression of MHC-II, CD40, CD80 and CD86 was measured by FACS. ΔMFI values are calculated by subtracting the mean fluorescence intensity (MFI) of the FMO control from the MFI of each sample **(D)**: n = 3. **(E)** CD4 T cells were cocultured with GM-CSF-driven BMDMs under Th17 polarizing condition as in **(A)**. IL-1β or IL-6 were added to the coculture and expression of IL-17A was analyzed by FACS. Shown are the representative FACS profiles with percentage of IL-17^+^ CD4 T cells obtained from two independent experiments. *p < 0.05; **p < 0.01; ***p < 0.001.

Dendritic cells capture antigens in the tissue and move into the draining lymph nodes to activate and polarize cognitive T cells into distinct types of helper T cells including Th1, Th17 and Treg cells (46, 47). In peripheral tissues, macrophages also regulate the functional activity of helper T cells by providing costimulatory ligands (*e.g.*, CD80, CD86, PD-L1/L2) and by secreting cytokines (IL-1β, IL-6, and IL-12) (21, 23, 24). To define the mechanistic as to how cpTLR-i modulates Th17 cells in the inflamed colonic tissues, we tested the effect of macrophages pretreated with cpTLR-i on the differentiation of CD4 T cells using a coculture system. M-CSF- or GM-SCF-derived BMDMs were treated with LPS in the presence or absence of cpTLR-i, and naïve CD4 T cells were activated and cocultured with the preactivated BMDMs. The results of Intracellular cytokine staining show that M-CSF-derived BMDMs preferentially leads to the polarization of CD4 T cells in IFN-γ-expressing Th1 cells but not in IL-17A-expressing Th17 cells (**Figure 7A**). We were able to induce IL-17A^+^ Th17 cells with GM-CSF-derived BMDMs in the presence of neutralizing antibodies to IL-12 and IFN-γ (**Figure 7B**). In this *in vitro* cocultivation, cpTLR-i-treated macrophages exhibited an impaired ability to induce the differentiation of CD4 T cells into Th17 cells (**Figure 7B**; **Supplementary Figure S5A**). We also found a relatively modest but significant attenuation in IFN-γ^+^ Th1 cell differentiation in the coculture with cpTLR-i-treated macrophages, although we did not observe a decrease in Th1 cells in the colonic tissues *in vivo* (**Figure 7A**; **Supplementary Figure S5B**). To test the possibility that the inhibition of TLR signaling could modulate CD4 cells intrinsically, we cultured CD4 T cells in the absence of macrophages under Th17 polarizing condition and found that cpTLR-i was not able to suppress Th17 cells intrinsically (**Supplementary Figure S5C**). Thus, we concluded that cpTLR-i suppresses the proinflammatory T cell-activating potential of macrophages, resulting in the compromised generation of Th17 cells.

To delineate how cpTLR-i impairs the ability of macrophages to help Th17 differentiation, we determined the induction of class II MHC molecules and costimulatory ligands. cpTLR-i treatment suppressed the surface expression of MHC-II and co-stimulators such as CD40, CD80, and CD86 in BMDMs activated with LPS (**Figures 7C, D**). Moreover, BMDMs treated with cpTLR-i secreted less amounts of cytokines IL-1β and IL-6, which have a role in promoting Th17 cell differentiation (48) shown in **Figure 1**. The addition of exogenous IL-1β to a Th17 coculture resulted in enhanced induction of IL-17A^+^ Th17 cells but that of IL-6 did not (**Figure 7E**). Of note, IL-1β addition partially restored the ability of cpTLR-i-treated macrophages to induce Th17 cell differentiation (**Figure 7E**; **Supplementary Figure S5D**). Overall, our experimental data suggest that TLR inhibition with cpTLR-i not only regulates the inflammatory responses of macrophages but attenuates Th17-mediated inflammation through the functional modulation of macrophages.

## Discussion

The intestinal tract not only needs to elicit a protective immune response against invading pathogens but also provide tolerance to symbiotic microbes (49). However, uncontrolled reactions to microbe-derived molecules such as TLR ligands may lead to chronic inflammatory conditions including IBD (50). Our transcriptomic analysis using macrophages provided evidence that the inhibition of TLR signaling by a cell-penetrating peptide could potentially modulate type 17 immune responses and IBD. Indeed, cpTLR-i was sufficient to mitigate colitis symptoms, colonic inflammation and tissue damage in mice given DSS. In agreement with the decreased Th17 responses *in vivo*, we found that the ability of macrophages to promote Th17 cells was substantially impaired by cpTLR-i in macrophage-CD4 T cell cocultures. On the other hand, cpTLR-i was not able to suppress the induction of Th17 cells in our *in vitro* culture with CD4 T cells alone, although other studies reported an intrinsic role of TLR signaling in Th17 cells and experimental autoimmune encephalomyelitis (51, 52). Further analyses revealed a decreased expression of IL-1β and costimulatory ligands such as CD80 and CD86 in macrophages treated with cpTLR-i, which could contribute to the defective T cell-supporting functions of macrophages (21, 53). Together, these results suggest that therapeutic targeting of the innate responses of macrophages to microbial products could be a strategy to circumvent the activation of pathogenic T cells in IBDs.

Macrophages exhibit a wide spectrum of phenotypes and the balance among functionally distinct macrophages is important for tissue homeostasis and pathology (54). In addition to decreased TNF-α^+^ macrophages, we observed increased Arginase-1^+^ macrophages in the colonic lamina propria of colitogenic mice treated with the cell-penetrating TLR inhibitor peptdie. Similarly, Jung et al. reported that inhibition of TLR2 dimer formation by a TLR2 trans-membrane peptide enabled the induction of macrophages associated with homeostatic or resident macrophage gene signatures while preventing that of macrophages with pro-inflammatory gene signatures in colitis mice (17). Thus, we wanted to determine whether cpTLR-i could regulate the functional activation of M2 macrophages with regard to the monocyte-macrophage waterfall (55). In our *in vitro* culture in the absence of a microbial TLR ligand, cpTLR-i did not affect the differentiation of monocytes into TNF-α^+^ macrophages and the production of proinflammatory cytokines such as TNF-α and IL-6. Intriguingly, the differentiation of TNF-α^+^ macrophages into Arginase-1^+^ cells with M2 phenotypes such as the expression of *Mrc1* and *Il10* was augmented by the cpTLR-i treatment. This finding was supported by the transcriptome analysis, in which the expression of genes associated with M2 macrophages, including *Osm*, *Gdf15* and *Vegfa*, were upregulated by cpTLR-i (**Figure 1H**) (31–33). Therefore, it is likely that TLR signaling negatively regulates the functional transition of M1 macrophages into M2 in a microbial TLR ligand-independent manner.

Given that cpTLR-i upregulates the functional maturation of M2 macrophages, we further defined a mechanism in which TLRs modulate signaling pathways downstream from M2 stimuli. Interestingly, the inhibition of TLR signaling by cpTLR-i enhanced the phosphorylation of Smad2/3 induced by TGF-β, an immunosuppressive cytokine. Studies have shown that TGF-β induces the expression of genes characteristic for alternatively activated macrophages while suppressing a pro-inflammatory phenotype which influences the functional activities of tumor-associated macrophages (56). Meanwhile, the activation of NF-κb by proinflammatory stimuli such as IL-1β was reported to inhibit TGF-β-induced gene expression by increasing the inhibitory Smad7 in hematopoietic progenitors and cancer cell lines (57, 58). In line with our observation that cpTLR-i could suppress IL-1β-induced phosphorylation of NF-κb p65 (**Figure 6G**), we speculated that the enhanced M2 differentiation could be partially due to attenuated negative regulation of the TGF-β signaling by suppressing the IL-1β-induced NF-κB activation. Thus, we propose a crosstalk between signaling pathways downstream of the TLRs and TGF-β receptors in the regulation of alternatively activated M2 macrophages although it remains to be further determined in the future studies.

Consistent with the functional modulation of mucosal immune responses, cpTLR-i was beneficial to the colonic epithelial integrity. However, we could not exclude the involvement of epithelial TLR signaling in the regulation of the mucosal barrier integrity. TLRs are expressed in the gut epithelium and enhance the intestinal barrier functions, including tightening of intracellular junctions, secretion of mucus and antimicrobial peptides and epithelial regeneration after injury (59). For example, stimulation with TLR2 or TLR4 agonist upregulates proliferative and anti-apoptotic factors including TFF3 in intestinal epithelial cells, whereas mice deficient for TLR2 or TLR4 have hypo-proliferative and pro-apoptotic phenotypes of the inflamed colons (3, 60). In agreement with our results, these studies suggest that TLRs could exert either a beneficial or detrimental effect on colitis, depending on which tissue compartments are engaged in the TLR signaling. Whether the intestinal epithelium is less sensitive to the cell-penetrating TLR inhibitor peptide needs to be further investigated. Of note, MyD88-independent induction of type I interferon responses such as the production of IFN-β was relatively less sensitive to cpTLR-i (**Figure 1** and data not shown). This corroborates the previous finding that *Ifnar1* ^-/-^ mice are more susceptible to colitis (61) and that IFN-β production triggered by double-stranded RNA of a commensal microbe protect mice from colitis (62).

TLRs are broadly expressed in the intestinal tissues and have a central role in controlling mucosal immune responses and maintaining the gut homeostasis, in responding to the microbial community. A growing amount of evidence suggests a potential use of therapeutic strategies that target TLR signaling in the prevention or treatment of autoimmune diseases such as IBD. However, only a few TLR agonists and antagonists are under clinical trials for therapeutic applications. In this study, our experimental data highlight the immunomodulatory potential of cpTLR-i and suggest this rationally designed cell-penetrating peptide as a novel therapeutic agent for IBD treatment. Future studies may explore an impact of the cell penetrating TLR inhibitor peptide on the gut barrier function in association with microbiota in the regulation of mucosal homeostasis.

## Methods

### Peptides, mice and colitis model

All peptides were provided by Genesen and the purity of each peptide was at least 95% as determined by HPLC fitted with a RP-C18 column (Phenomenex). The amino acid sequence of cpTLR-i is KLALKLALKALKAALKLASHCRVLLI. The sequences of CP and TLR-i control peptides are KLALKLALKALKAALKLA and SHCRVLLI, respectively. C57BL/6J mice were purchased from DBL and maintained under specific pathogen-free conditions at the Laboratory Animal Resource Center of Hallym University. Mice were allowed to acclimate for at least one week to minimize variability in the gut microbiota composition and randomly divided into experimental groups. Eight-week-old male C57BL/6J mice were given drinking water containing 2.5% dextran sodium sulfate (MP Biomedicals) for 7 days and normal water for the remainder of the days. Mice were injected intraperitoneally with 16 mg/Kg cpTLR-i or control peptides every other day from day -2 to day +6. The disease activity index was scored as described previously (34): body weight loss (‘0’: no loss; ‘1’: loss > 1-5%; ‘2’: loss > 5-10%; ‘3’: loss > 10-20%; ‘4’: loss > 20%); stool consistency (‘0’: firm; ‘2’: loose; ‘4’: diarrhea), and presence of blood (‘0’: absence; ‘1’: hemoccult; ‘2’ hemoccult with visual pellet bleeding; ‘4’: gross bleeding, blood around the anus). Scores were added to give a maximum score of 12.

### Macrophage depletion

Macrophage were depleted by injecting intravenously with 200 μl of liposomes containing clodronate (Encapsula Nanosciences) three times (day -1, +2 and +5) in accordance to the manufacturer’s instructions.

### Preparation of bone marrow-derived macrophages and cocultivation with CD4 T cells

BMDMs were prepared as described previously (63). Briefly, bone marrow cells were isolated and grown in IMDM media supplemented with 10% FBS in the presence of 20 ng/ml M-CSF (PeproTech) or 5 ng/ml GM-CSF (PeproTech) for 7 days. BMDMs were pretreated with cp-TLR-i for 1 h and activated with 200 ng/ml LPS (Sigma) for the indicated times. For cocultivation with T cells, CD4 T cells were purified from the spleen using anti-CD4 microbeads and MACS column (Miltenyi Biotec). For Th1 polarization, M-CSF-derived BMDMs were treated with 200 ng/ml LPS for 6 h, washed twice with fresh IMDM containing 10% FBS, and cocultured with CD4 T cells in the presence of 2.5 μg/ml anti-CD3ε (BD Biosciences). GM-CSF-driven BMDMs were activated with LPS and cocultured with CD4 T cells in the presence of anti-CD3ε and neutralizing antibodies against IL-12 and IFN-γ (BD Biosciences) to induce Th17 polarization. Four days after the cocultivation, CD4 T cells were restimulated with 50 ng/ml PMA and 1 μg/ml ionomycin for 6 h or with 0.5 μg/ml anti-CD3e and 0.5 μg/ml CD28 for 1 day for intracellular cytokine staining and cytokine beads array, respectively.

### Cell viability assay and NO measurement

BMDMs were pretreated with the indicated concentrations of cpTLR-i and activated with 200 ng/ml LPS for 24 h. Cell viability was measured using the EZ-Cytox Enhance Cell Viability Assay Kit (DoGenBio) in accordance to the manufacturer’s instruction. Production of nitrogen oxide (NO) in the culture supernatant was determined by the Griess Reagent System (Promega).

### RAW 264.7 cell culture and transfection

RAW 264.7 macrophage cell line was obtained from Hyung-Joo Kwon (Hallym University) and maintained in DMEM supplemented with 10% FBS. RAW 264.7 cells were transfected with pCRIII-WT-MYD88, pCRIII-DN-MyD88, pRK5-WT-TRAF6, pRK5-DN-TRAF6, or control vector using the FuGene HD transfection reagent (Promega) in accordance to the manufacturer’s instructions. A day after transfection, cells were pretreated with 2 μM cp-TLR-i for 1 h and activated with 200 ng/ml LPS (Sigma) for 8 h.

### Confocal microscopy

BMDMs were seeded on a chamber slide and incubated with 2 μM cpTLR-i or control peptide for the indicated times. Cells were washed three times with ice-cold PBS and fixed with 4% paraformaldehyde. After permeabilized with 0.2% Triton X-100, cells were stained with monoclonal antibody against the TLR antagonist peptide overnight at 4°C followed by secondary antibody conjugated with Alexa Fluor 647 (ThermoFisher). The coverslip was mounted using a mounting solution containing DAPI (ThermoFisher) and images were taken using a Zeiss LSM710 laser scanning confocal device attached to an Axiovert 100 microscope (Carl Zeiss).

### Cytokine measurement and colon explants

Amounts of cytokines in the culture supernatants were analyzed by cytokine beads array (CBA) with the LEGENDplex™ Mouse Inflammation panel (BioLegend) and Th1/Th2/Th17 CBA kit (BD Biosciences) in accordance to the manufacturer’s instructions. Colonic tissue explant samples, approximately 10 mm in length, were isolated from the distal region of the colon. The colon explants were washed in PBS, turned inside out, and incubated in 500 μl IMDM supplemented with 10% FBS at 37°C. After a 24-h incubation, culture supernatants of the colon explants were centrifuged at 14,000 x g for 10 min to remove debris and analyzed by CBA. Amounts of IL-18 was determined using mouse IL-18 ELISA kit (MBL Life Science). Cytokine levels were normalized to the weight of each tissue sample.

### Transcriptomic analysis

Total RNA was purified from BMDMs using TRIzol reagent (Invitrogen) and the cDNA library was prepared with the TruSeq Standard mRNA Library Prep Kit (Illumina, San Diego, CA, USA) following the manufacturer’s instructions. The libraries were sequenced using a NovaSeq 6000 platform (Illumina) by Macrogen. Qualified reads were mapped to the *Mus musculus* mm10 reference genome by HISTA2 version 2.1.0. and assembled using StringTie version 2.1.3b. Differentially expressed genes (DEGs) were identified with DESeq2, and a more than 1.5-fold change with a p value less than 0.05 was considered statistically significant. Functional annotation of DEGs was performed with DAVID (https://david.ncifcrf.gov), and the statistically enriched pathway was analyzed using the KEGG database in DAVID. A heat map of the relative expression pattern of the DEGs was created with the heatmap.2 function of the gplot package in RStudio version 3.4.4.

### Fecal sample ELISA

Fecal pellets were collected 5 days after the administration of DSS and weighed. A single fecal pellet was homogenized in 200 μl PBS and centrifuged at 10,000 rpm for 5 min to remove the debris. The amounts of Lipocalin-2 and Calprotectin in the fecal supernatant were measured with the Mouse Lipocalin-2/NGAL and S100A8/S100A9 Heterodimer detection kits (R&D Systems), respectively. The levels of Lipocalin-2 and Calprotectin were normalized to the weight of each fecal pellet.

### Histology

Mouse colons were collected and rinsed with ice-cold PBS. Samples were fixed with 4% paraformaldehyde, dehydrated, and embedded in paraffin. For histopathologic analysis, the distal colon tissue sections were stained with hematoxylin and Eosin Y solution (Merck). The sections were incubated with 1% Alcian Blue 8GX (Sigma) in 3% acetic acid for 15 min and then with 0.5% periodic acid solution (Sigma) for 5 min and Schiff’s reagent (Sigma) for 15 min to stain goblet cells.

### Isolation of the colonic lamina propria cells and flow cytometry

Inflammatory cells were isolated from the colonic lamina propria as described previously (34). Briefly, colons were washed with ice-cold PBS, inverted, and incubated in RPMI1640 containing 1 μM DTT, 0.6 mM EDTA and 1% FBS for 30 min in a 37°C shaking incubator. After washing with fresh media, the colon was cut into pieces and placed in RPMI1640 containing 25 μg/ml Liberase TL (Roche), 25 μg/ml Liberase DL (Roche) and 125 μg/ml DNase I (Sigma). After a 30-min incubation with gentle shaking, the cell suspension was washed with fresh media and passed through a 70 μm cell strainer. Immunofluorescence staining was conducted as described previously (63). A single cell suspension isolated from the colonic lamina propria or *in vitro* culture was stained with antibodies against surface markers for 30 min on ice. Fluorophore conjugated antibodies against CD11b (clone M1/70), Ly6G (clone 1A8), CD64 (clone X54-5/7.1), CD3ϵ(clone 145-2C11), CD4 (clone RM4-5), CD8 (clone 53-6.7), I-A/I-E (clone M5114.15.2), F4/80 (clone 6F12), CD80 (clone 16-10A1) and CD86 (clone GL1) were purchased from BD Biosciences. Anti-TCR-β (clone H57-597) and anti-Ly6C (clone HK1.4) were obtained from ThermoFisher. Anti-TCR γ/δ (clone GL3) and anti-CD40 (clone 3123) from BioLegend. For intracellular staining, cells were restimulated with 50 ng/ml PMA and 1 μg/ml ionomycin in the presence of the Gogi-Stop and Golgi-Plug reagent (BD Bioscience) for 6 h. After fixation and permeabilization, the cells were stained with fluorescence-labelled antibodies to TNF-α (clone MP6-XT22, BD Bioscience), IFN-γ (clone XMG1.2, BD Biosciences), IL-17A (clone ebio17B7, ThermoFisher) and Arginase 1 (clone AlexF5, ThermoFisher). FoxP3 intracellular staining was performed with the mouse FoxP3 staining set (ThermoFisher). Data were acquired using the FACS Canto II instrument with the FACSDiva software (BD Bioscience) and analyzed by FlowJo V10 software (BD Bioscience).

### Monocyte isolation and culture

Monocytes were purified from bone marrow cells with the Monocyte Isolation Kit (Miltenyi Biotec) and MACS columns following the manufacturer’s instructions. Purified monocytes were activated with 5 ng/ml M-CSF, 50 ng/ml IFN-γ and 10 ng/ml IL-23 and cultured for 3 days in the presence of 2 μM cpTLR-i or vehicle, to give rise to macrophages with a proinflammatory immature phenotype. Then, the *in vitro* generated macrophages were further cultured with 5 ng/ml TGF-β and 10 ng/ml IL-4 in the presence of cpTLR-i or vehicle for an additional 3 days to be differentiated into macrophages with a mature resident phenotype. Monocyte-derived macrophages were restimulated with 50 ng/ml PMA and 1 μg/ml ionomycin in the presence of the Gogi-Stop and Golgi-Plug reagent (BD Bioscience) and stained for surface markers. After fixation and permeabilization, cells were stained intracellularly with PE-conjugated anti-TNF-α and APC-labelled anti-Arginase-1 for flow cytometry. Culture supernatants were analyzed by CBA.

### Quantitative real-time PCR and western blotting

RNA was extracted from BMDMs with the TRIzol Reagent (Invitrogen) and reverse transcribed into cDNA by the ImProm-II Reverse Transcription system (Promega). Quantitative PCR was performed with an SYBR PCR mix (Toyobo) and the CFX Real-Time PCR detection system (Bio-Rad), using the primer pairs listed in **Supplementary Table S1**. For Western blotting, the cells were lysed in 20 mM HEPES (pH 7.4), 150 mM NaCl, 1% Triton X-100 and 1 mM EDTA supplemented with a protease inhibitor and phosphatase inhibitor cocktail (GenDEPOT) and applied for SDS-PAGE. Immunoblotting was performed with the indicated primary antibody followed by the anti-rabbit secondary antibody conjugated with HRP. All primary antibodies were purchased from Cell Signaling Technology except anti-FLAG monoclonal antibody (Sigma). Protein bands were detected on X-ray film using an Enhanced Chemiluminescence kit (Merck Millipore).

### Statistical analysis

All experiments were performed in more than triplicate and the results of two or three independent experiments were represented as the mean (± S.D). Differences between the samples were analyzed using an unpaired, two-tailed Student’s t-test with the Prism software version 7.04 (GraphPad Software, San Diego, CA, US). Two-way ANOVA with Bonferroni correction was applied to analyze the body weight loss and disease activity index using Prism. Statistical significance was denoted as follows: *, p < 0.05; **, p < 0.01; *** p < 0.001.

## Data availability statement

The RNA-seq data have been deposited at the GENE Expression Omnibus (GEO) under accession number GSE224903.

## Ethics statement

The animal study was reviewed and approved by Institutional Animal Care and Use Committee of Hallym University.

## Author contributions

Conceptualization, KL and SL. Data curation, BT, SP and KL. Investigation, BT, SP, DC and KL. Resources: HS and SL. Writing-original draft, BT, SP and KL. Supervision, KL. All authors contributed to the article and approved the submitted version.
