# Supplementary material for: Cell-penetrating TLR inhibitor peptide alleviates ulcerative colitis by the functional modulation of macrophages
Source: Front Immunol. 2023 May 5;14:1165667. doi: 10.3389/fimmu.2023.1165667 (PMC10196052; doi:10.3389/fimmu.2023.1165667)

SUPPLEMENTAL INFORMATION

Cell-penetrating TLR inhibitor peptide alleviates ulcerative colitis by the functional modulation of macrophages

Bikash Thapa[1†], Seongwon Pak[2†], Dohyeon Chung[2], Hye Kyoung Shin[3], Seong Ho Lee[3] and Keunwook Lee[1,2*]

[1]Institute of Bioscience & Biotechnology, , Hallym University, Chuncheon, Republic of Korea, [2]Department of Biomedical Science, Hallym University, Chuncheon, Republic of Korea, [3]R&D Center, Genesen Co., Ltd, Seoul, Republic of Korea

*Correspondence: Keunwook Lee, Ph.D. Email. keunwook@hallym.ac.kr

†These authors contributed equally

Supplemental Table S1. Primer sequences used in the quantitative real-time PCR analysis.

Gene name	Sequence (5' to 3')
Actb	Forward: GGCACCACACCTTCTACAATG<br>Reverse: GGGGTGTTGAAGGTCTCAAAC
Arg1	Forward: GGGACCTGGCCTTTGTTGAT<br>Reverse: GCTTCCAACTGCCAGACTGT
Mrc1	Forward: GCTTCCGTCACCCTGTATGC<br>Reverse: TCATCCGTGGTTCCATAGACC
Chil3	Forward: GAAGGAGCCACTGAGGTCTG<br>Reverse: TGAGCCACTGAGCCTTCAAC
Retnla	Forward: AGTGCCCTGTGTTTCAGAGA<br>Reverse: TGTGGAAGTTCACGCTCCAG
Il10	Forward: CCCATTCCTCGTCACGATCTC<br>Reverse: TCAGACTGGTTTGGGATAGGTTT

Supplemental Figure Legend

Figure S1. cpTLR-i modulates the proinflammatory activation of macrophages associated with type 17 immune responses and inflammatory bowel diseases. (a) BMBDs were pretreated with 10 μM brefeldin A, 1 μM rotenone or vehicle for 1 h and incubated in the presence of 2 μM cpTLR-i for 30 min. Shown are the representative confocal images stained with antibody against the TLR inhibitor peptide. (b) BMDMs, B cells preactivated with LPS, $CD4^+$ T cells preactivated with anti-CD3 and anti-CD28 antibodies, and colorectal cancer cell line SW480 were cultured with cpTLR-i as in (a) and analyzed by confocal microscopy. (c-e) BMDMs were pretreated with 2 μM cpTLR-i or control peptides (TLR-i and CP peptides) and activated with LPS as in Figure 1. Cell viability (c) and NO production (d) were determined 24 h after LPS treatment and amounts of TNF-α in the culture supernatant (e) were measured by CBA. (f) The heatmaps for genes associated with IL-17 signaling pathway, Th17 cell differentiation and inflammatory bowel disease are shown.

Figure S2. Therapeutic potential of cpTLR-i in colitis animals depends on the cell-penetrating ability of the peptide. (a-c) Mice were injected intraperitoneally with the control TLR-i peptide lacking the cell-penetrating conjugate (TLR-i) or CP peptide and given DSS as in Figure 2. (a) Body weight was measured daily and (b) disease activity index was scored. (c) The colons were isolated 9 d after starting the DSS administration and their lengths were measured.

Figure S3. cpTLR-i attenuates infiltration of inflammatory leukocytes into the colonic mucosa. Lamina propria cells in the colons were analyzed as in Figure 4 and numbers of $CD45^+$ (whole leukocytes), TCR-β^+ $CD4^+$ (CD4 T cells), $CD11b^+$ $Ly6G^+$ (neutrophils/granulocytes) and $CD11b^+$ $Ly6G^-$ $CD64^+$ (monocytes/macrophages) populations are shown.

Figure S4. Effect of cpTLR-i on the expression of TGF-β target genes. BMDMs pretreated with cpTLR-i or vehicle were activated with LPS for 2 h and mRNA expression of *Id1*, *Smad7* and *Dap2ip* were analyzed by RNA sequencing as in Figure 1.

Figure S5. cpTLR-i attenuates Th17 responses through the modulation of macrophages. (a, b) CD4 T cells were cocultured with LPS-activated BMDMs under Th17 (a) and Th1 (b) polarization conditions as in Figure 7. 4 d after the cocultivation, CD4 T cells were restimulated with anti-CD3 and anti-CD28 antibodies and the culture supernatants were analyzed by CBA. (c) CD4 T cells were pretreated with cpTLR-i and cultured with anti-CD3 and anti-CD28 antibodies in the absence of BMDMs under Th17 polarizing condition. Shown are the representative FACS profiles in the viable $CD4^+$ T cell gate and bar graph indicating percentages of IL-$17A^+$ CD4 T cells. (d) CD4 T cells were cocultured with BMDMs and IL-1β or IL-6 was added to the coculture as in Figure 7E. 4 d after the cocultivation, secretion of IL-17A from the activated T cells was measured by CBA.

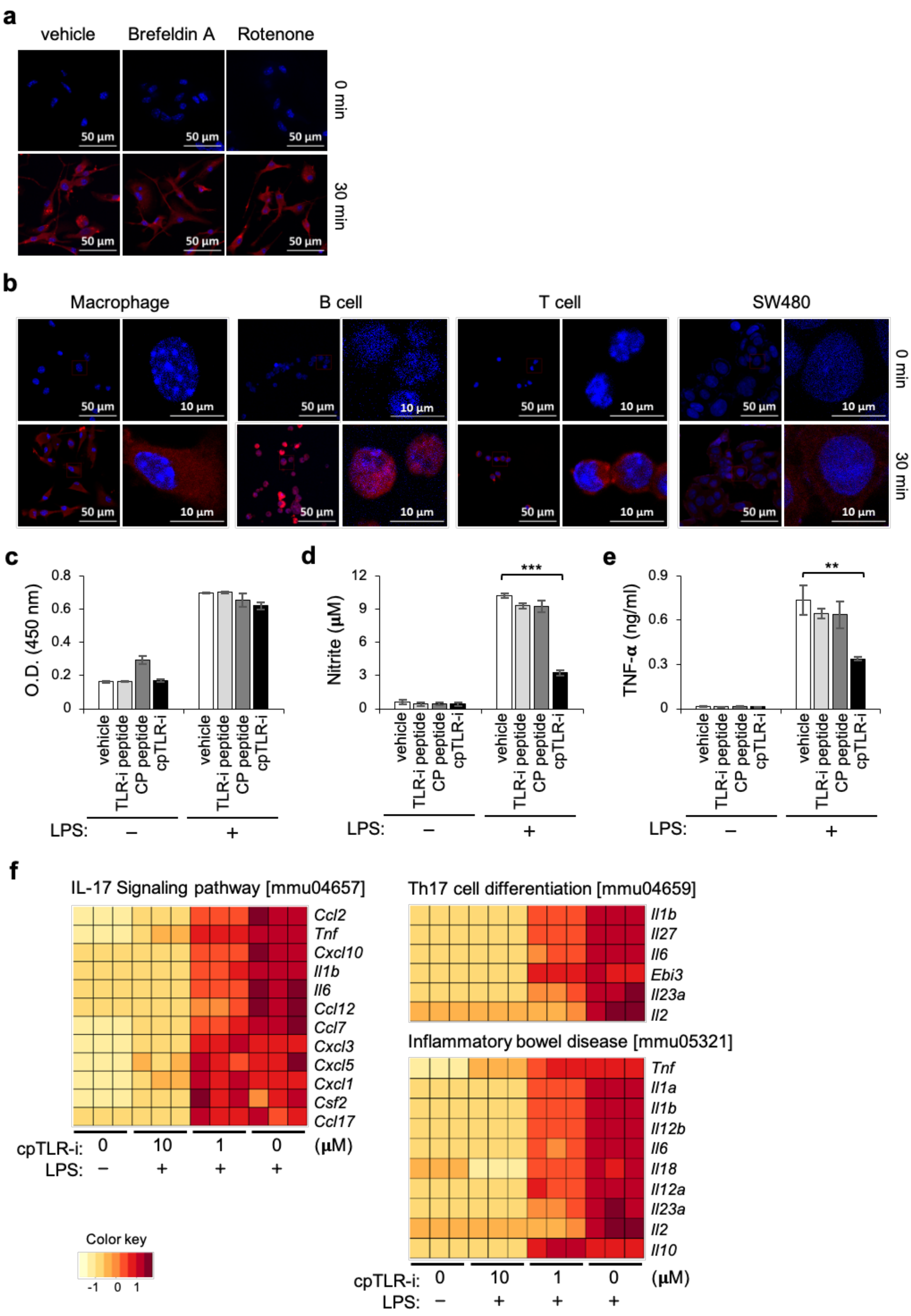
a
vehicle
Brefeldin A
Rotenone
0 min
30 min
50 µm
b
Macrophage
B cell
T cell
SW480
10 µm
c
O.D. (450 nm)
d
Nitrite (µM)
e
TNF-α (ng/ml)
vehicle
TLR-i peptide
CP peptide
cpTLR-i
LPS:
***
**
f
IL-17 Signaling pathway [mmu04657]
Ccl2
Tnf
Cxcl10
Il1b
Il6
Ccl12
Ccl7
Cxcl3
Cxcl5
Cxcl1
Csf2
Ccl17
Th17 cell differentiation [mmu04659]
Il27
Ebi3
Il23a
Il2
Inflammatory bowel disease [mmu05321]
Il1a
Il12b
Il18
Il12a
Il10
cpTLR-i:
(µM)
Color key

Thapa & Pak *et al*. Supplemental Figure S2

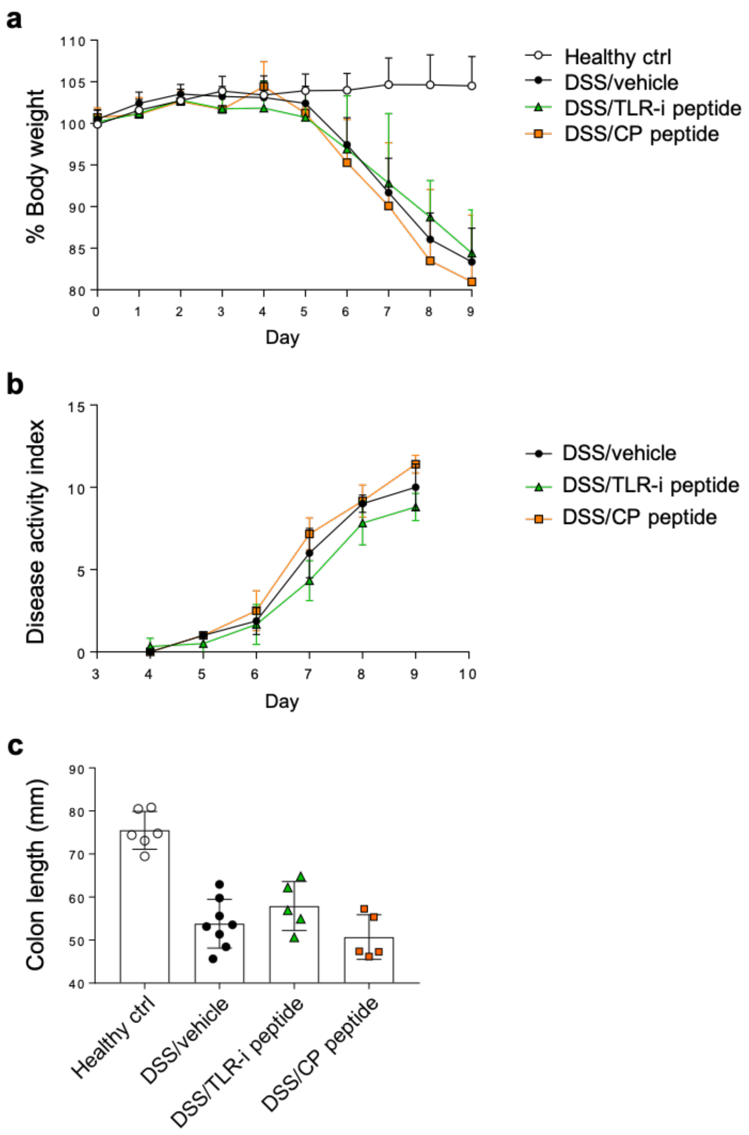

Thapa & Pak *et al*. Supplemental Figure S3

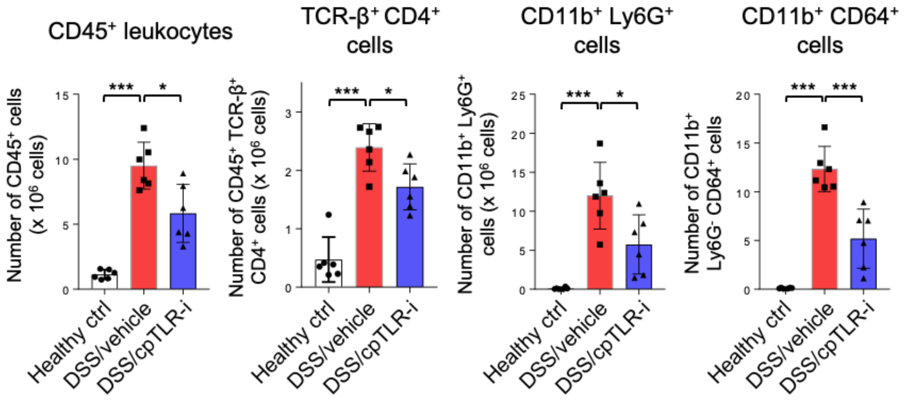

Thapa & Pak *et al*. Supplemental Figure S4

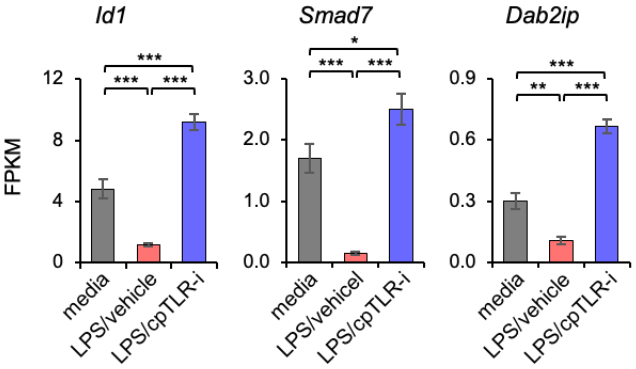

Thapa & Pak *et al*. Supplemental Figure S5

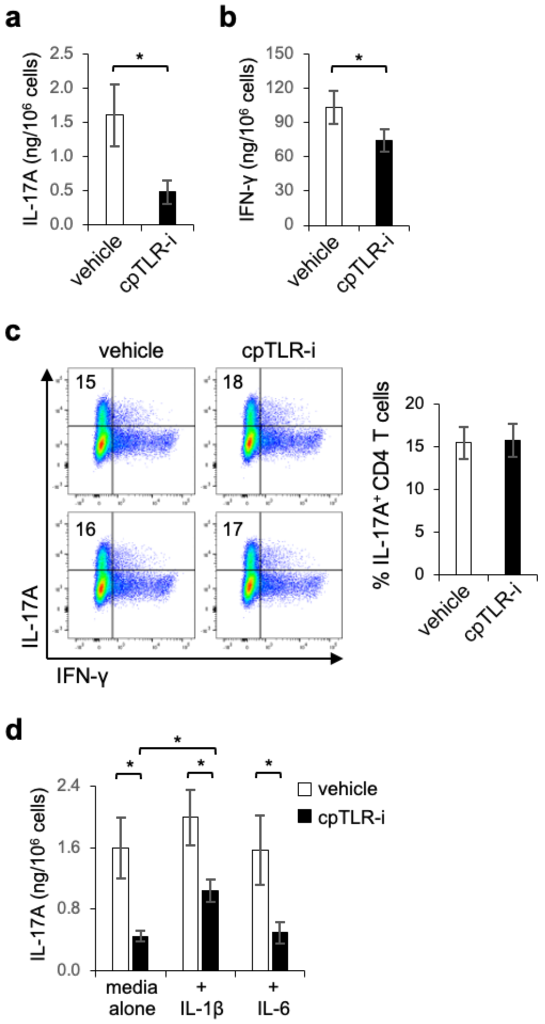

Supplement: Supplementary file 1 [file DataSheet_1.pdf]
